# Oligomeric Receptor Complexes and Their Allosteric Receptor-Receptor Interactions in the Plasma Membrane Represent a New Biological Principle for Integration of Signals in the CNS

**DOI:** 10.3389/fnmol.2019.00230

**Published:** 2019-09-25

**Authors:** Dasiel O. Borroto-Escuela, Kjell Fuxe

**Affiliations:** ^1^Department of Neuroscience, Karolinska Institutet, Stockholm, Sweden; ^2^Department of Biomolecular Science, Section of Physiology, University of Urbino, Campus Scientifico Enrico Mattei, Urbino, Italy; ^3^Grupo Bohío-Estudio, Observatorio Cubano de Neurociencias, Yaguajay, Cuba

**Keywords:** G protein-coupled receptor, heteroreceptor complexes, oligomerization, brain disorders, allosteric receptor-receptor interactions, allosteric dynamic, dopamine receptor, dimerization

## Abstract

G protein-coupled receptors (GPCRs) not only exist as monomers but also as homomers and heteromers in which allosteric receptor-receptor interactions take place, modulating the functions of the participating GPCR protomers. GPCRs can also form heteroreceptor complexes with ionotropic receptors and receptor tyrosine kinases modulating their function. Furthermore, adaptor proteins interact with receptor protomers and modulate their interactions. The state of the art is that the allosteric receptor-receptor interactions are reciprocal, highly dynamic and substantially alter the signaling, trafficking, recognition and pharmacology of the participating protomers. The pattern of changes appears to be unique for each heteromer and can favor antagonistic or facilitatory interactions or switch the G protein coupling from e.g., Gi/o to Gq or to beta-arrestin signaling. It lends a new dimension to molecular integration in the nervous system. Future direction should be aimed at determining the receptor interface involving building models of selected heterodimers. This will make design of interface-interfering peptides that specifically disrupt the heterodimer possible. This will help to determine the functional role of the allosteric receptor-receptor interactions as well as the integration of signals at the plasma membrane by the heteroreceptor complexes, vs. integration of the intracellular signaling pathways. Integration of signals also at the plasma membrane seems crucial in view of the hypothesis that learning and memory at a molecular level takes place by reorganization of homo and heteroreceptor complexes in the postsynaptic membrane. Homo and heteroreceptor complexes are in balance with each other, and their disbalance is linked to disease. Targeting heteroreceptor complexes represents a novel strategy for the treatment of brain disorders.

## Summary of the Established Principles. GPCR Homomers and Heteromers and Their Allosteric Receptor-Receptor and Receptor-Protein Interactions

In 1980–1981, we introduced the concept that peptide and monoamine signals in the Central Nervous System (CNS) could become integrated in the plasma membrane through direct peptide receptor-monoamine receptor interactions (Agnati et al., 1980; Fuxe et al., 1981, 1983). It was based on the findings that specific peptides could modulate the binding characteristics, especially the affinity, of the monoamine receptors in membrane preparations in a receptor subtype specific manner (Fuxe et al., 1983). In 1993 the hypothesis was introduced that direct allosteric receptor-receptor interactions became possible through the formation of G protein-coupled receptor (GPCR) heterodimers in balance with the corresponding homodimers (Zoli et al., 1993). The receptor-receptor interactions can involve alterations in recognition, pharmacology, signaling and trafficking of the partner receptor protomers (Fuxe and Agnati, 1985; Fuxe et al., 1998, 2010a).

In 1997, the cloning of the gene encoding the Gamma-aminobutyric acid (GABA) B receptor 1 (GABABR1) was obtained (Kaupmann et al., 1997). However, this GABABR1 did not reach the plasma membrane but remained on the intracellular membranes with a low affinity for GABAB receptor agonists. In 1998, Marshall and colleagues (White et al., 1998) made the highly significant discovery that heterodimerization was crucial for the formation of a functional GABA B receptor. Thus, they found a new subtype of GABAB receptor, namely GABABR2 that physically interacted with GABABR1 *via* their intracellular carboxy-terminal regions using the yeast two-hybrid system. This interface had the structure of a coiled-coil domain which led to the transport of the GABABR1 to the plasma membrane with transformation of the GABABR1 protomer into a state with a high affinity for GABA. The functional response of the GABAB heterodimer was mediated by the GABABR2 protomer coupled to Gi/o as studied inter alia in *Xenopus oocytes* possessing G protein-coupled inwardly-rectifying potassium channels (GIRKs; White et al., 1998). This important discovery had a strong impact on the heterodimer field and their receptor-receptor interactions.

In 1998, the discovery was also made that receptor activity-modifying proteins (RAMPs) can regulate the family B GPCR calcitonin-receptor-like receptor (CLR) in terms of transport to the plasma membrane and recognition specificity (McLatchie et al., 1998). The RAMPs represent a family of proteins with a single transmembrane domain, RAMP1, RAMP2 and RAMP3 (McLatchie et al., 1998). Upon association of CLR with RAMP1 it becomes a calcitonin gene related peptide (CGRP) receptor. Instead, association with RAMP2 and RAMP3 changes the CLR into adrenomedullin1 and adrenomedullin 2 receptors (McLatchie et al., 1998; Barwell et al., 2012; Hay and Pioszak, 2016; Hay et al., 2016). Adrenomedullin is a potent vasodilator (Muff et al., 1995; Poyner, 1997).

The diversity was further increased by demonstrating that when the calcitonin receptor becomes associated with RAMP3 it changes to recognize amylin with high affinity and becomes an amylin receptor, named amylin 3 receptor (Christopoulos et al., 1999; Hay et al., 2016). Instead, when associated with RAMP1 the calcitonin receptor alters its recognition to recognize both amylin and CGRP with high affinity and becomes an amylin receptor named amylin 1 receptor (Christopoulos et al., 1999; Hay and Pioszak, 2016). Amylin is known to inhibit both insulin release and food intake. Altogether this impressive work underlines the dynamic regulation of the CGRP and calcitonin receptors by plasma membrane proteins and its impact on trafficking, recognition and signaling of these GCPRs. As discussed in the article of Hay et al. (2016), it seems likely that RAMPs can interact with a large number of class B GPCRs and certain class A and C GPCRs leading to allosteric changes in receptor recognition, signaling and trafficking of the participating GPCRs.

**Figure 1:**
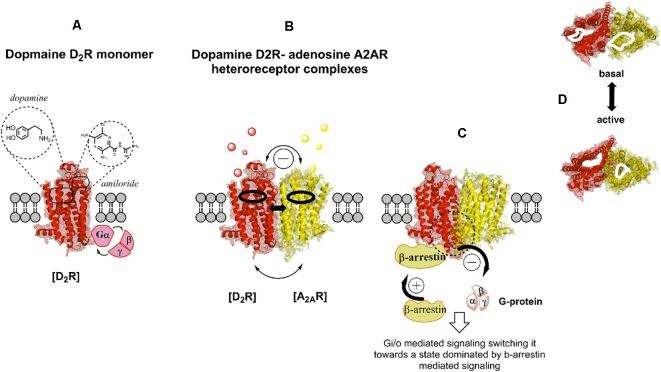
Illustration of the G protein-coupled receptor (GPCR) allosteric modulation from receptor monomers to heteroreceptor complexes through an example of the dopamine D2R-adenosine A2AR (D2R-A2AR) heteroreceptor complex. Studies on negative co-operativity and neuropeptide-monoamine receptor–receptor interactions in the central nervous system (CNS) in the early 1980s opened up the potential existence of receptor dimers and receptor mosaics together with receptor monomers **(A)**. Homo- and hetero-receptor complexes increases the diversity of GPCR recognition and their signaling. Upon activation of one protomer modulation of the orthosteric and allosteric binding sites of the adjacent protomer take place **(B,C)** as well as of its G protein activation and selectivity, and therefore its signaling cascades. For instance, among other changes a switch from G protein to β-arrestin signaling may take place **(C)**. **(D)** Top view of the orthosteric binding pocket for each protomer in the A2AR-D2R heteroreceptor complexes before and upon co-activation. Allosteric receptor-receptor interactions can take place on the respective orthosteric binding sites for each protomer. The allosteric modulation operates through conformational changes in the interface interactions of these homo and heteroreceptor complexes located in the transmembrane domain of each protomer.

Adaptor/chaperone proteins like Sigma1R that bind to the receptor protomers can also substantially modulate the receptor-receptor interactions (Pinton et al., 2015b; Beggiato et al., 2017; Borroto-Escuela et al., 2017b). Allosteric mechanisms were proposed to mediate the reciprocal receptor-receptor interactions *via* the receptor interface of homo-heteroreceptor complexes over which the allosteric waves passed based on the early work on allosteric transitions in the 1960s (Monod et al., 1965; Koshland et al., 1966), updated in 2009 (Tsai et al., 2009). The allosteric receptor-receptor interactions take place when the binding of a ligand to an orthosteric site or an allosteric site induces a conformational change in the partner receptor protomer after passing the receptor interface (see Figure 1). Multiple allosteric changes can be induced in the partner receptor with, for example, changes in affinity and/or signaling efficacy. In case of allosteric enhancement of orthosteric ligand binding in receptor heteromers the terms positive cooperativity or agonistic allosteric modulation are often used, while in case of allosteric antagonism negative cooperativity or antagonistic allosteric modulation can be used (see Fuxe and Agnati, 1987; Dasgupta et al., 1996; Christopoulos and Kenakin, 2002; Han et al., 2009; Borroto-Escuela et al., 2010a,c; Fuxe et al., 2010a,b; Cottet et al., 2013). Heterodimerization of somatostatin and opioid receptors was also found to allosterically modulate phosphorylation, trafficking and desensitization of the receptor protomers (Pfeiffer et al., 2002).

**Figure 2:**
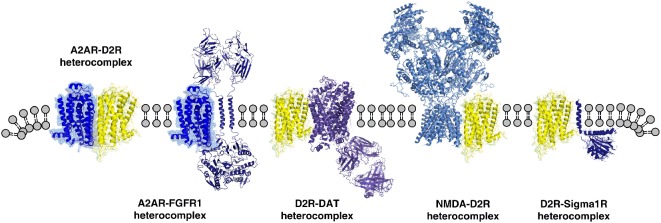
Illustration of the diversity of heteroreceptor complexes that are formed between different families of receptors in the plasma membrane shown as heterodimers. The A2AR-D2R heterodimer is shown as an example GPCR-GPCR heteromers (Borroto-Escuela et al., 2013c, 2018b,d). The A2AR-FGFR1 heterodimer (Flajolet et al., 2008; Borroto-Escuela et al., 2013b) is shown as an example of GPCR-RTK heteromers. The D2R-N-Methyl-D-aspartate (NMDA) heterodimer (Liu et al., 2006) is shown as an example of GPCR-ionotropic receptor heteromers. D2R-dopamine transporter (DAT) complex (Lee et al., 2007) is shown as an example of GPCR-monoamine transporter complexes. The D2R-Sigma1 receptor heterodimer is shown as an example of GPCR-single spanning transmembrane receptor heteromer (Pinton et al., 2015b; Borroto-Escuela et al., 2016a, 2017b).

Therefore, allosterism in GPCR has an impact at the intracellular signaling level (Luttrell and Kenakin, 2011; Mahoney and Sunahara, 2016). Here, the allosteric receptor-receptor interaction can modulate the strength of the receptor-G protein coupling or switch the G protein coupling from one type of G protein to another type of G protein as is the case in the D1R-D2R heteromer (George et al., 2014). It is also known that GPCR kinases (GRK) can assist GPCRs in switching from G protein mediated signaling to beta-arrestin mediated signaling (Komolov and Benovic, 2018). It seems likely that allosteric A2AR-D2R interactions (Figure 2) can participate in such events since they were shown to inhibit D2R Gi/o mediated signaling and increase D2R mediated beta-arrestin signaling through recruitment of beta-arrestin to the intracellular surface of the D2R (Borroto-Escuela et al., 2011b; Figure 1).

## The Class A GPCR Heteromers

### The Example of Multiple Adenosine A2AR Heteromers and Their Clinical Relevance

A large number of A2AR heteromers exist and represent a hub component in the GPCR heterodimer network (GPCR-HetNet^1^; Borroto-Escuela et al., 2017a). The A2AR heteromers can be listed as follows: A1R-A2AR, A2AR-A2BR, A2AR-D2R, A2AR-D3R, A2AR-D4R4. They are usually named A2AR homo, iso, and heteroreceptor complexes in view of the participation of adaptor/chaperone proteins directly binding to the GPCR protomers (Borroto-Escuela and Fuxe, 2019). The A2AR-D2R heteroreceptor complex is of high interest in view of its relevance for Parkinson’s disease, Schizophrenia and cocaine addiction (Fuxe et al., 2014d, 2015; George et al., 2014; Borroto-Escuela et al., 2018d). This receptor complex is characterized by a dynamic antagonistic allosteric receptor-receptor interaction by which the A2AR agonist can inhibit the D2R protomer recognition, and the Gi/o mediated signaling of the D2R protomer which instead becomes coupled to beta-arrestin 2. In the dorsal striatum the A2AR-D2R heteroreceptor complexes are in balance mainly with A2A homoreceptor complexes and A2AR monomers mainly located on the dorsal striatopallidal GABA neurons mediating motor inhibition. The inhibitory D2R signaling is, therefore, necessary to reduce activity in this neuronal system and allows movements to develop. In agreement, the A2AR antagonists produce antiparkinsonian actions which can involve the blockade of the A2AR protomer in the A2AR-D2R complex, especially in view of the reduction of the extracellular DA levels in Parkinson’s disease (Surmeier et al., 2014).

It is, however, clear that the antiparkinsonian effects of A2AR antagonists were only modest in clinical trials of patients with Parkinson’s disease (Navarro et al., 2016). One explanation may be that in the early synthesis of A2AR antagonists the knowledge of the molecular mechanism for human A2AR ligand binding and activation was insufficient. However, this is no longer true as shown in a recent review (Carpenter and Lebon, 2017). The structure of the A2AR is now well characterized at the atomic level using both X-ray crystallography and cryo-EM (García-Nafría et al., 2018). The binding mode of A2AR antagonists (Doré et al., 2011; Cheng et al., 2017) and A2AR agonists (Lebon et al., 2011) have been compared with the characterization of the agonist-induced conformational changes. It should be underlined that structure-based drug design was used to develop novel types of A2AR antagonists. It involved the discovery by Dr. Marshall’s group of 1,2,4- Triazine derivatives as A2AR antagonists, which could enter and bind deeply in the orthosteric binding pocket (Congreve et al., 2012). This work was based on structures of thermostabilized A2A receptors. Different types of A2A receptor ligands could be designed through a platform approach based on structure (Congreve et al., 2012). Protocols for the generation of optimally thermostabilized membrane proteins for structural studies were recently published with a focus on mutagenesis and screening strategy (Magnani et al., 2016).

Should the novel A2AR antagonists not improve the treatment of Parkinson’s disease vs. the old A2AR antagonists, another hypothesis can be provided for the lack of strong antiparkinsonian actions of the novel A2AR antagonists. In the development of Parkinson’s disease there may be reorganization of the A2AR-D2R heteroreceptor complexes as reductions of the extracellular DA levels develop. Based on such a reorganization the A2AR protomer may, for example, reduce its affinity for the A2AR antagonist in the novel A2AR-D2R complex formed. Thus, the A2AR orthosteric binding pocket may be altered through the reorganization. The reorganization may also involve an increased recruitment of mGluR5 to the A2AR-D2R complex which leads to an enhancement of the allosteric inhibition of the D2R protomer signaling in the A2AR-D2R-mGluR5 complex (Fuxe et al., 2003; Antonelli et al., 2006; Borroto-Escuela and Fuxe, 2019). Based on such a view, it may be important to provide the A2AR antagonist early in the treatment of Parkinson’s disease before reorganization of the A2AR-D2R complex. This may also reduce the affinity of the A2AR and D2R for each other and instead the formation of D2R homomers and monomers can be favored with improvement of treatment (Borroto-Escuela and Fuxe, 2019).

Future hopes include performing crystallization of the A2AR-D2R dimer involving the selection of different conformational states. It should include structural determinants of A2AR and D2R ligand binding in this heterodimer, which may lead to novel A2A receptor antagonist targeting the A2AR protomer in the A2AR-D2R heterodimer.

### A2AR—Alpha-Synuclein Interaction and Its Relevance for the Development of Parkinson’s Disease

It is of high interest that alpha-synuclein can produce an increase in A2AR signaling that can enhance toxicity and aggregation of alpha-synuclein (Ferreira et al., 2017). These effects are blocked by an A2A receptor antagonist which may be related to the observation that agonists of A2AR can enhance the calcium passage over N-methyl-D-aspartate receptors (NMDARs; Rebola et al., 2008). It is proposed that monomeric alpha synuclein transmembrane peptides become associated with A2AR complexes and modulate their signaling. We propose that the A2AR antagonist may enhance the synthesis of an alpha-synuclein dimer that does not propagate, while the A2AR agonist in the A2AR-alph-synuclein complex produce signals that enhance the propagation of alpha-synuclein dimers into large synuclein aggregations. As a result, A2A receptor signaling may contribute to neurodegeneration in Parkinson’s disease (Borroto-Escuela and Fuxe, 2019).

A putative A2AR-D2R-NMDAR complex may also participate in these neurodegenerative events. The alpha synuclein monomer may bind to the A2AR protomer of this receptor complex and enhance the A2AR activation, with an increase in the allosteric inhibition of the D2R. In this way, the NMDAR signaling is freed from the D2R inhibition (Liu et al., 2006). As a consequence, the NMDAR increases its signaling with an increased flow of calcium ions over the NMDAR channels, which can be linked to nitric oxide production and help explain the mechanism for the neurodegeneration linked to alpha-synuclein-A2AR interactions in the striatopallidal GABA neurons.

The hypothesis is introduced that the alpha synuclein fibrils formed in the dendrites and soma of the dorsal A2AR positive striatopallidal GABA neurons can reach the vulnerable DA nerve terminals *via* extracellular vesicle-mediated volume transmission (Borroto-Escuela et al., 2018a; Borroto-Escuela and Fuxe, 2019). The alpha-synuclein fibrils formed in the dendrites and soma of the striatopallidal neurons can accumulate in, for example, exosomes released into the extracellular fluid and internalized into the surrounding DA nerve terminals. This can start the onset of the degeneration of the vulnerable DA nerve terminals. Later on, through retrograde transport of the alpha-synuclein fibrils the degeneration process can start in the vulnerable nigral DA cell bodies. Previous work has indicated that the degeneration of the vulnerable nigrostriatal DA neurons begin in the striatal DA nerve terminal networks (Surmeier et al., 2017). For the first time, it seems possible to understand how the degeneration of the nigrostriatal DA neurons can become linked to the appearance of A2AR induced alpha-synuclein fibrils in the striatopallidal GABA neurons and their transport to the striatal DA terminals *via* extracellular vesicle-mediated volume transmission. The hypothesis emphasizes how distinct GPCRs can play a significant role in building up large protein molecules, like alpha-synuclein aggregations, to which the nigrostriatal DA neurons are highly vulnerable to and from which Parkinson’s disease develops. It seems possible that the amyloid hypothesis of Alzheimer’s disease can also involve distinct GPCR induced changes in the fragmentation of the amyloid precursor protein. The beta amyloid fragment appears to play a fundamental role in the formation of senile plaques in the extracellular space, that interfere with volume transmission in the extracellular space and the modulation of synaptic transmission, and thus with brain communication.

### A2AR Antagonists and Their Relevance for Cancer Immunotherapy

Sitkovsky and colleagues (Kjaergaard et al., 2018) demonstrated that gene deletion of the A2AR, as well as the A2AR antagonist, removes the tumor-induced immunosuppression of the tumor-reactive CD8^+^ T cells. Thus, the immunosuppression involves the activation of A2AR located on the T cells. In line with these results, an A2AR antagonist produces similar results and is associated with an increased secretion of IFN-gamma from the CD8^+^ T cells (Yuan et al., 2017). A hypoxia-adenosine driven immunosuppression appears to be involved in the activation of A2AR on the T cells. The detailed mechanism is unknown, but it has been speculated that A2AR can be part of a chemokine/cytokine heteroreceptor complex, in which the A2AR protomer upon activation, puts a brake on chemokine/cytokine receptor signaling releasing, for example, IFN-gamma. In support of this view, the A2AR protomer is known to put a brake on D2R signaling in the A2AR-D2R complex (Borroto-Escuela et al., 2018d). IL1R2, CCR2, and CXCR4 may also form heteroreceptor complexes with NMDAR and D2R, which is of relevance for the mild neuroinflammation hypothesis of Schizophrenia (Borroto-Escuela et al., 2017c). Therefore, there is the possibility that A2R activation produces its immunosuppressant actions through receptor-receptor interactions in A2AR-Chemokine/cytokine complexes on the T cell membrane. It should be mentioned that extensive formation of heteromers may also exist between opioid and chemokine receptor subtypes (Tarakanov and Fuxe, 2015).

However, drug development in the chemokine receptor field has most likely been limited due to a lack of structural information. Chemokine receptor type 9 (CCR9) is of special interest since its activation leads to recruitment of leucocytes to the gut and CCR9 is a target for the treatment of Chron’s disease in which massive inflammation develops in the intestinal tract. It should, therefore, be noted that Marshall and colleagues (Oswald et al., 2016) could establish the crystal structure of CCR9 when in complex with an intracellular allosteric CCR9 antagonist. Allosteric CCR9 binding sites in the transmembrane bundle when occupied by an allosteric receptor antagonist can offer treatment of Chron’s disease through interference with the G protein coupling of the CCR9 receptor.

The Marshall group also very recently determined the structure of the compliment C5a receptor bound to the extra-helical antagonist, NDT9513727 (Robertson et al., 2018). The complementary cascades are obtained upon activation and produces anaphylatoxins like C5a. It produces proinflammatory actions *via* its GPCR anaphylatoxin chemotactic receptor (C5aR1) located on cells of myeloid origin (Woodruff et al., 2011). It is of high significance that the structure of the C5aR1 complexed with NDT9513727 contains a negative allosteric binding pocket located in an extrahelical position. The NDT9513727 is considered to be a wedge that blocks the transmembrane (TM5) movements that leads to the inhibition of C5aR1 signaling with reduction of inflammation. A new strategy for the treatment of inflammation has been obtained, including neuroinflammation, from the excellent work completed by Marshall and colleagues.

### The Nicotinic Acid Receptor

The ligand for this receptor is nicotinic acid, better known as vitamin B3, and is used as an antidyslipidemic drug with a highly significant impact (Offermanns, 2006). Its receptor is a GPCR receptor of class A. Upon activation it is known to lower low-density lipoprotein cholesterol levels and increase high-density lipoprotein cholesterol levels. It was discovered to be a GPCR receptor by three groups in 2003 (Soga et al., 2003; Tunaru et al., 2003; Wise et al., 2003). This receptor is termed GPR109A and is coupled to Gi proteins and located inter alia on adipocytes and immune cells. It was quite an achievement to identify the nicotinic acid receptor as a GPCR. Class A GPCRs also moved into the field of metabolism regulation representing novel therapeutic targets in this area.

An important side-effect of nicotinic acid is the induction of cutaneous flushing *via* GPR109A activation (Goldsmith and Cordill, 1943). It was discovered by Lefkowitz and colleagues that GPCRs can also signal *via* beta-arrestins which can bind to the intracellular interface of GPCRs (Dewire et al., 2007) in competition with GPCRs. In an excellent article by the Lefkowitz group (Walters et al., 2009), it was discovered that beta-arrestin 1 is responsible for the flushing induced by nicotinic acid but not for the antilipolytic actions. The mechanism involves the beta-arrestin 1 mediated activation of the cytosolic phospholipase A_2_, with the formation of the prostaglandin D_2_ precursor, leading to vasodilation which causes the flushing. These important observations open up the possibility to develop G protein biased drugs that can avoid the induction of flushing. It seems possible that this can be accomplished through a ligand induced formation of GPR109A heteroreceptor complexes, in which the receptor partner can favor the G protein biased signaling. In case of the A2AR-D2R complexes the A2AR agonist does the opposite and favors the beta-arrestin signaling of the D2R (Fuxe et al., 2014d).

## The Class B GPCR Heteromers

This family of GPCRs shows a substantially different structure compared with class A. In 2008, it became evident that the members of this family, also called the secretin like family, mainly interacted with each other as studied with BRET in cellular models (Harikumar et al., 2008). A large number of class B receptors were, for example, found to heterodimerize with the secretin receptor. Later on, it was observed with BRET that the ligand glucagon-like peptide 1 can produce heterodimerization between the gastric inhibitory peptide (GIP) receptor and the glucagon-like peptide 1 receptor belonging to class B receptors (Schelshorn et al., 2012). Today we know the structure of the human transmembrane domain in the glucagon receptor as well as in complex with the antagonist MK-0893 (Jazayeri et al., 2016). It is of high interest that Marshall and colleagues could discover a new allosteric binding pocket in the class B receptor located in an extra-helical position.

In 2007, it was found that a class B receptor can, in fact, form a heteromer with a class A receptor (Young et al., 2007). In cellular models it was demonstrated, with BRET, that the vasopressin V1b receptor and corticotropin releasing hormone type1 (CRHR1) can form a constitutive heterodimer. In continued research, it was observed in 2012 that the V1b-CRHR1 heterodimer mediates the synergistic functional interactions observed after cotreatment with CRH and vasopressin. There was an inter alia enhancement of the CRH induced cAMP accumulation by vasopressin in HEK293 cells (Murat et al., 2012). In further cellular research, it was established that CRH type 2 alpha can physically interact with D1R to form heteroreceptor complexes in living cells. It switched D1R protomer signaling into increasing intracellular calcium levels upon activation (Fuenzalida et al., 2014), which may have relevance for the modulation of addiction. In line with these findings, it was observed that overexpression of CRHR1 in calcium-calmodulin-dependent kinase II neurons of the central amygdala, enhanced vulnerability towards alcohol reinstatement behavior (Broccoli et al., 2018). This work demonstrates the class B receptor CRHR1 and CRHR 2alpha can, in fact, form a heteroreceptor complex with a class A receptor, namely the D1R.

It is of high interest that the crystal structure of the transmembrane domain of the CRHR1 has been established in complex with a small molecule antagonist CP 376395 (Hollenstein et al., 2013). This non-peptide ligand appears to bind deeply in the CRHR1, to act as an allosteric receptor antagonist to produce anti-stress effects offering new targets for drug development in anxiety. In the future, it would be great to know the structure of the CRHR1-D1R heteromer to understand what happens to the orthosteric and allosteric binding pockets and the allosteric receptor-receptor interactions over the receptor interface.

## The Class C GPCR Heteromers

### Metabotropic Glutamate Receptor Heteroreceptor Complexes

The most well-known members of this class are the mGlu receptors and the GABAB receptors (Pin et al., 2005). A major feature of this class of receptors is their existence as dimers. As an interesting example, the eight metabotropic glutamate receptors will be discussed (Conn and Pin, 1997). Their structure have special features characterized by a special and large extracellular domain involving the venus fly trap, to which the glutamate transmitter binds. A domain enriched in cysteine, which is linked to the transmembrane domain, also exists. It is of high interest that the crystal structure of the transmembrane domain of the mGluR5 has recently been obtained, increasing our understanding of its architecture with allosteric binding sites. Identification of key micro-switches regulating the selection of receptor signaling was also obtained (Doré et al., 2014).

The mGlu receptors can be divided into three groups based on the similarity of their sequence, their transduction, and their pharmacology. Subgroup I consists of mGluR1 and mGluR5 which signal *via* Gq/11, resulting in the activation of the PLC-beta pathway involving the stimulation of PKC and increased intracellular levels of calcium *via* formation of IP3. Their stimulation also produces depolarization leading to increased excitability. The mGluR1 and mGluR5 are located in a postsynaptic location, mainly in an extrasynaptic position. The subgroup II consists of mGluR2 and mGluR3 and mGluR 4, 6, 7, and 8 form subgroup III. They all signal *via* G_i/o_ proteins, which inhibit adenylate cyclase activity and voltage gated calcium channels, inhibiting the release of neurotransmitters. Postsynaptically they can open up inwardly rectifying potassium channels leading to hyperpolarization.

A large number of GPCR interacting proteins participate in the mGluR regulation of all subgroups (Fagni et al., 2000; Bockaert et al., 2010). As an example, it may be mentioned that mGluR5 is associated with neuronal Ca2+ binding protein 2 (Canela et al., 2009) and forms a coassembly and coupling with SK2 channels (Calcium activated potassium channels; García-Negredo et al., 2014). A protein-mGluR5 interaction of special interest is the mGluR5 interaction with contactin-associated protein 1 since it appears crucial for the ability of mGluR5 to control memory formation in the hippocampus (Morató et al., 2018).

The studies on class C mGluR subtypes in the brain, demonstrate that they not only form heteroreceptor complexes among each other but also with multiple class A GPCRs (Borroto-Escuela et al., 2014). It indicates that several mGluR subtypes can play a significant integrative role in synaptic and extra-synaptic glutamate transmission, by participating in multiple heteroreceptor complexes of relevance for brain diseases and their treatment.

### mGluR1-mGluR5 Heteroreceptor Complexes

This receptor complex was identified in the cerebral cortex and hippocampus based on an interaction proteomics strategy using knockout controls (Pandya et al., 2016). Super resolution microscopy also demonstrated their existence in the hippocampal neurons (Pandya et al., 2016). Furthermore, this receptor complex seems to be in balance with the corresponding homo-receptor complexes in glutamate synapses of the hippocampus in both synaptic and extrasynaptic locations (Jong et al., 2014). The composition of the mGluR1-mGluR5 complexes is unknown but there are indications that scaffolding proteins, phosphatases and kinases can participate (Hayashi et al., 2009). It is still unknown if these receptor complexes exist in the direct and indirect pathways of the basal ganglia, in spite of their colocation in these neurons (Tallaksen-Greene et al., 1998).

### mGluR2-mGluR4 Heteroreceptor Complexes

These heteroreceptor complexes were found using a new technique to study plasma membrane receptor complexes and also involved time resolved FRET (Doumazane et al., 2011). Further work in 2012 (Kammermeier, 2012) established that the mGluR2-mGluR4 complexes could function in neurons, while combined treatment, but no single treatment with mGluR2 and mGluR4 agonists, led to the activation of the heteroreceptor complex (Kammermeier, 2012). As to negative and positive allosteric modulator binding sites (NAM and PAM), two NAMs and a single PAM exists on each of the receptor protomers. Activation of both NAMs in each receptor protomer is needed to inhibit the signaling of this receptor complex. In the case of PAM activation, it is of interest that selective PAMs failed to increase the glutamate signaling of this receptor complex, while selective PAMs did so in the mGluR1-mGluR5 receptor complex (Goudet et al., 2005). It seems possible that each mGluR receptor complex possesses its own sets of NAMs and PAMs, established by structure and modulated by allosteric receptor-receptor and receptor-protein interactions (see also Pin et al., 2005). A unique and similar pharmacology of mGluR2-mGluR4 heteroreceptor complexes appears to exist in cellular models and in striatum (Conn et al., 2014; Yin and Niswender, 2014). However, further work is needed with a proximity ligation assay to establish their modulation of brain circuits.

This is also true for putative mGluR4-mGluR7 heteroreceptor complexes which show similar distribution patterns in the striatum and pallidum (Bradley et al., 1999; Kosinski et al., 1999).

### mGluR1-A1R Heteroreceptor Complexes

In 2001, this heteroreceptor complex was discovered by (Ciruela et al., 2001) based on experiments with coimmunoprecipitation. The analysis was performed in HEK 293 cells and in cerebellar synaptosomes. The receptor-receptor interactions were found to be subtype-specific. It was the first time a mGluR was indicated to interact with a GPCR that was not an isoreceptor. In the functional receptor-receptor interactions a synergy was demonstrated following agonist coactivation as to intracellular calcium signaling (Ciruela et al., 2001).

In recent years further support was given for the existence of mGluR1-A1R heteroreceptor complexes based on an analysis of non-neuronal cells using coimmunoprecipitation and FRET (Kamikubo et al., 2015). Indications for antagonistic functional receptor-receptor interactions were, in this case, obtained. Thus, activation of mGluR1 produced a reduction of inhibitory Gi/o mediated A1R signaling on adenylate cyclase (Kamikubo et al., 2015). It was proposed that such an action by mGluR1 can contribute to cerebellar long-term depression, known to involve mGluR1 activation.

### Putative mGluR1-GABA(B) Heteroreceptor Complexes

mGluR1 and GABA(B) receptors are located in an extra synaptic position on dendrites of Purkinje cells (Hirono et al., 2001). Activation of GABA(B) receptors enhanced the mGluR1-induced actions present in a juxtaposition to parallel fiber-Purkinje cell glutamate synapses (Hirono et al., 2001). GABA released from surrounding GABA interneurons may reach and activate the extrasynaptic GABA(B) receptors *via* short distance volume transmission. The enhancement of mGluR1 function may take place *via* the existence of a mGluR1-GABA(B) heteroreceptor complex on the Purkinje cell dendrites with allosteric receptor-receptor interactions through which the GABA(B) receptor increases the mGluR1 signaling. However, evidence for this molecular mechanism remains to be obtained.

### mGluR5-A2AR and mGluR5-D2R Heteroreceptor Complexes

In 2001, it was for the first time possible to obtain clear-cut indications for the existence of mGluR5-A2AR heteroreceptor complexes based on neurochemical and behavioral findings on mGluR5-A2AR receptor-receptor interactions (Popoli et al., 2001). In biochemical binding experiments, it was observed that the mGluR5 agonist CHPG produced an enhancement of the inhibitory actions of the A2AR agonist CGS 21680 on the high affinity component of the D2R agonist binding sites in striatal membranes. It was matched by the ability of the mGluR5 agonist to enhance the inhibitory actions of the A2AR agonist on D2R agonist-induced contralateral turning behavior in a hemi-parkinsonian rat (Popoli et al., 2001). In 2002, structural support for this view was obtained a demonstration of mGluR5-A2AR heteroreceptor complexes in cellular models upon cotransfection and in membrane preparations from rat striatum involving coimmunoprecipitation (Ferré et al., 2002). As to the effects on the heteromer signaling of this complex, coactivation of the two receptor protomers led to a synergistic increase of ERK phosphorylation and to inhibition of motor activity induced by phencyclidine The existence of mGluR5-D2R heteroreceptor complexes was demonstrated for the first time in 2009 by Ciruela and colleagues by means of bimolecular fluorescence complementation (Cabello et al., 2009). The results obtained are in line with the results obtained in biochemical binding studies on mGluR5 agonist modulation of the affinity of the high affinity state of the D2R agonist binding sites in striatal membrane preparations (Popoli et al., 2001).

### A2AR-D2R-mGluR5 Heteroreceptor Complexes

A major result was obtained by the Ciruela group in 2009 (Cabello et al., 2009), providing evidence for the existence of trimeric A2AR-D2R-mGluR5 heteroreceptor complexes in living cells. It became possible through the combined use of bimolecular fluorescence complementation with BRET or sequential BRET-FRET. In the striatum, these heterotrimeric complexes appeared to be present in a juxtaposition to striatal glutamate synapses in the plasma membrane based on high-resolution immunoelectron microscopy (Cabello et al., 2009). The existence of these trimeric complexes was supported by coimmunoprecipitation in homogenates of the striatum. Their stoichiometry and spatial distribution remain to be determined. It seems possible that the allosteric receptor-receptor interactions in these higher order complexes can reorganize, to reduce and recognize D2R signaling more effectively in the striatopallidal GABA neurons. It can be a highly dynamic process. It represents an important heteroreceptor complex that should be targeted for the treatment of Parkinson’s disease, schizophrenia and cocaine addiction.

There are strong indications that A2AR-D2R and A2AR-mGluR5 heteroreceptor complexes exist on the glutamate terminals, forming synapses on the striatopallidal GABA neurons (Rodrigues et al., 2005; Ciruela et al., 2006; Tozzi et al., 2007; Shen et al., 2008). It is therefore clear that trimeric A2AR-D2R-mGluR heteroreceptor complexes can also participate in the regulation of the glutamate release process. Thus, integration at the presynaptic levels of the glutamate synapses also plays a relevant role in motor function.

With the increasing disappearance of the DA terminals and extracellular DA levels, especially in the striatum of Parkinson’s disease, a major reorganization of the multiple A2AR and mGluR5 complexes at the pre and postsynaptic level of the cortico-striatal glutamate synapses will take place, with marked disturbances in motor function.

A2AR and mGluR5 antagonists should be especially effective to produce antiparkinsonian actions in view of their increasing dominance, since the inhibitory D2Rs lose their function (Schwarzschild et al., 2006). Increases in extracellular levels of adenosine and glutamate will develop due to reduced inhibition exerted by D2R. This may enhance the formation of A2AR-D2R, mGluR5-D2R and the trimeric A2AR-D2R-mGluR heteroreceptor complexes which leads to further inhibition.

Against this background, the wearing off of the antiparkinsonian actions of levodopa and D2R agonists can be related to an increase in the brake of D2R protomer signaling, produced by the allosteric inhibition exerted by activated A2AR and mGluR5 protomers in the reorganized receptor complexes. It also involves a marked disappearance of the D2R monomers and homodimers since they are recruited into various A2AR and mGluR heteroreceptor complexes.

### mGluR5-MOR Heteroreceptor Complexes

These heteroreceptor complexes were demonstrated using coimmunoprecipitation in HEK 293 cells (Schröder et al., 2009). The negative allosteric receptor modulator mGluR5 MPEP was found to alter the allosteric receptor-receptor interactions and in this way produce modulation in the MOR agonist-induced phosphorylation, internalization and desensitization of the MOR protomer in these complexes in the HEK 293 cells. These results open the possible relevant role of such receptor complexes in the brain, especially in pain and addiction.

### mGluR2-5-HT2AR Heteroreceptor Complexes

This heteroreceptor complex was identified in 2008 and appeared to be involved in psychosis (González-Maeso et al., 2008). Subsequent work established that three alanine residues in transmembrane IV of mGluR2 played a key role in the receptor interface (Moreno et al., 2012). In the same year, the existence of the mGluR2-5-HT2A receptor complex was validated using time-resolved FRET (Delille et al., 2012). Its relevance for the cellular signaling cascades was also demonstrated.

In the late 1990s, it was indicated that the 5-HT2A receptors in the primate cerebral cortex, especially in the apical dendrites of the pyramidal nerve cells, could be targets for hallucinogenic and antipsychotic drugs (Jakab and Goldman-Rakic, 1998). It is therefore of interest that hallucinogenic but not standard 5-HT2A agonists can recruit Gi/o mediated signaling to cortical 5-HT2A receptors, resulting in inhibition of the AC-PKA pathway associated with changes in behavior (González-Maeso et al., 2007). It was proposed in 2009 that one major target for hallucinogenic drugs was the mGluR2-5-HT2A heteroreceptor complex located in pyramidal neurons of the cerebral cortex (González-Maeso and Sealfon, 2009).

It should be considered that the ability of the hallucinogenic 5-HT2A agonists to recruit Gi/o mediated signaling pathways (González-Maeso et al., 2007) can involve a unique biased action at the orthosteric binding site of the 5-HT2A protomer of the mGluR2-5-HT2A heteroreceptor complex. This action can produce an altered allosteric receptor-receptor interaction which enhances the Gi/o mediated signaling of the mGluR2 protomer with increased inhibition of adenylyl cyclase activity. It may be proposed that combined stimulation of the Gq mediated signaling (5-HT2AR protomer) and enhanced Gi/o mediated signaling (mGluR2 protomer) can have pathological effects, blocking the correct depolarization and hyperpolarization patterns in large numbers of cortical pyramidal neurons. Such effects can lead to dysfunctions which can result in hallucinations and psychosis.

However, it should be noticed that the Gi/o mediated effects of hallucinogenic 5-HT2A receptor agonists could not be validated (Delille et al., 2012). More experiments should, therefore, be performed to understand if the mGluR2-5-HT2A heteroreceptor complex is a relevant target for hallucinogenic 5-HT2A agonists and the molecular mechanisms involved. Furthermore, it was not possible to validate (Delille et al., 2012) that the hallucinogenic 5-HT2A agonist DOI increases the affinity of the mGluR2 agonist binding sites (Moreno et al., 2012). It amplifies the need for further work.

Besides allosteric mechanisms in receptor heteromers, the physiological antagonism demonstrated between 5-HT2A receptors and mGluR2, for example, in the prefrontal cortex (Marek et al., 2000) can also involve posttranslational modifications like protein phosphorylation (Mao et al., 2011) as well as interactions in the intracellular signaling pathways.

Finally, it should be mentioned that mGluR2-5-HT2B heteroreceptor complexes have also been demonstrated using homogenous time-resolved FRET (Delille et al., 2012). Their existence and function in the brain are unknown.

## GPCR-Ionotropic Receptor Heteromers

In 2000, evidence was obtained that GPCR can also form heteromers with ionotropic receptors (Liu et al., 2000). Thus, D5R-GABA(A)R receptor complexes were demonstrated with the D5 carboxy-terminal domain interacting with the second intracellular loop of the GABA(A) gamma2(short) receptor subunit. Inhibitory reciprocal allosteric receptor-receptor interactions developed involving a dynamic modulation of synaptic strength in the GABA(A) ion channels (Liu et al., 2000). A couple of years earlier GABA(A) receptor activation had been found, by our group, to reduce the affinity of the high affinity D2R agonist binding sites in membrane preparations from the dorsal striatum (Pérez de la Mora et al., 1997). These results indicated the existence of allosteric receptor-receptor interactions in D2R-GABA(A)R complexes.

In 2002, Dr. Fang Liu and colleagues also found the existence of D1R-N-Methyl-D-aspartate (NMDA) receptor complexes. They demonstrated that two regions of the D1R carboxyl tail couple to the NMDA glutamate receptor subunits NR1-1a and NR2 (Lee et al., 2002). The dual regulation of the NMDAR involved inhibition of its currents and reduction of excitotoxicity through an inositol trisphosphate (IP3)/Ca^2+^ mechanism. We had previously observed that L-Glutamate reduced the high affinity D2R agonist binding sites in striatal membranes using the [^3^H]N-propylnorapomorphine radioligand (Fuxe et al., 1984). In 2006, a direct D2R-NR2B subunit interaction was observed in the postsynaptic membrane of glutamate synapses on striatal neurons (Liu et al., 2006). This interaction blocks the association of Ca^2+^/calmodulin-dependent protein kinase II (CaMKII) with the NR2B subunit, reduces its phosphorylation and produces inhibition of NMDAR currents. As a result, D2R activation *via* cocaine-induced dopamine (DA) release can bring down activity in the ventral striatopallidal GABA neurons, mediating anti-reward. It seems possible that a reciprocal allosteric antagonistic D2R-NMDAR interaction can exist in these neurons (Figure 2).

## GPCR-Receptor Tyrosine Kinase Heteromers

In 2007, we introduced the hypothesis of the existence of GPCR-receptor tyrosine kinase (RTK) heteroreceptor complexes based on their potential direct receptor-receptor interactions (Fuxe et al., 2007). More precisely, the FGFR1-5-HT1AR heteromer was proposed to exist. The following year a direct physical interaction was demonstrated between FGFR1 and A2AR (Figure 2; Flajolet et al., 2008; Borroto-Escuela et al., 2013b). FGF was found to act as a cotransmitter to the A2AR protomer to enhance synaptic plasticity at the morphological and functional level. The FGFR1-5-HT1AR heteroreceptor complexes were found in 2012 (Borroto-Escuela et al., 2012, 2013a, 2016b) and produced an enhancement of hippocampal plasticity. This heteroreceptor complex was proposed to be a novel target for anti-depressant drugs at which neurotropic and antidepressant actions can be induced, by restoring tropism and activity in the neuronal hippocampal circuits, showing deficits in depression (Borroto-Escuela et al., 2016b).

In 2017, it was also found that FGFR can form heteroreceptor complexes with muscarinic acetylcholine receptors (mAChR) associated with increased neurite outgrowth in neural hippocampal cultures (Di Liberto et al., 2017). However, it should be considered that sometimes the GPCR and RTK trophic interactions only involve a functional crosstalk in the intracellular pathways for receptor transactivation to develop, as is the case for the GABAB and insulin growth factor 1 interaction (Tu et al., 2010).

## Adaptor Proteins as Part of Receptor Complexes With Focus on Sigma1r as a Target for Cocaine

Of special interest in the case of adaptor proteins is the sigma-1 receptor (Sigma1R) that also acts as an intracellular chaperone and is located at the endoplasmic reticulum-mitochondria interface (Kourrich et al., 2012). In addition, cocaine acts as an agonist at Sigma1R. Through the cocaine agonist action, Sigma1R can be translocated to the plasma membrane where it forms a complex inter alia with D1R and D2R receptors and modulates their signaling (Navarro et al., 2010, 2013; Pinton et al., 2015b). However, Sigma1R does not bind to the D3R and D4R (Navarro et al., 2013). Findings indicate that the Sigma1R may bind to multiple sites at the D2R since the BRET signal from the Sigma1R-D2R complex is only weakly reduced by Sigma1R in increasing concentrations (Figure 2; Pinton et al., 2015a,b; Borroto-Escuela et al., 2016a).

It is of substantial interest that in Sigma1R-D2R complexes cocaine (100 nM) can markedly increase its ability to enhance D2R Gi/o mediated inhibition of the CREB signal in cotransfected cells vs. D2R singly transfected cells (Pinton et al., 2015a,b). This action was associated with a marked reduction in the cocaine-induced internalization of the D2R. These findings were validated in studies on striatal synaptosomes involving glutamate and dopamine release under enhanced inhibitory D2R modulation through cocaine acting at Sigma1R-D2R complexes (Ferraro et al., 2012; Beggiato et al., 2017). It increases our understanding of the acute actions of cocaine, in which D2R-Sigma1R complexes can significantly participate.

In the case of cocaine self-administration, a different but exciting story develops (Borroto-Escuela et al., 2018d). The Sigma1R expression level becomes preferentially increased in the nucleus accumbens (Romieu et al., 2002; Borroto-Escuela et al., 2017b) and an increase in A2AR-D2R and D2R-Sigma1R heteroreceptor complexes was observed in the nucleus accumbens shell (Borroto-Escuela et al., 2017b). This led to increased antagonistic A2AR-D2R interactions with reduced affinity of the D2R high affinity agonist binding sites (Pintsuk et al., 2016; Borroto-Escuela et al., 2018d). It was previously found in cellular models that cotransfection of Sigma1R, A2AR and D2R cDNAs led to marked enhancement of the cocaine and A2AR agonist-induced inhibition of D2R Gi/o mediated signaling (Pinton et al., 2015a,b). An A2AR-D2R-Sigma1R complex was postulated to be formed with significant enhancement of the antagonistic A2AR-D2R receptor-receptor interactions. It was proposed that in cocaine addiction A2AR-D2R-Sigma1R can be formed in a nucleus accumbens shell with a permanent brake on D2R signaling through altered allosteric receptor-receptor interactions (Borroto-Escuela et al., 2018d). It is a fine indication how adaptor protein Sigma1R can strongly enhance the antagonistic allosteric A2AR-D2R receptor-receptor interactions in the A2AR-D2R-Sigma1R complex.

These results also offer novel strategies for future treatment of cocaine addiction based on the removal of pathological A2AR-D2R-Sigma1R complexes with a strong brake on D2R Gi/o mediated signaling and a reduction of the D2R affinity (Borroto-Escuela et al., 2018d). Interface-interfering peptide A2AR TM5 may be of high value since it can disrupt the A2AR-D2R heteroreceptor complex and enhance cocaine self-administration in rats (Borroto-Escuela et al., 2018e). A rat A2AR synthetic TM5 peptide was used and found to effectively bring down the BRET1 signal of A2AR-D2R heteromers in HEK293 cells. Upon intra-accumbal microinjections of A2AR-TM5, significant reductions were observed in the number of A2AR-D2R PLA clusters per cell in the sampled field in the nucleus accumbens shell and core, but not in the dorsal striatum. The antagonistic A2AR-D2R receptor-receptor interaction was counteracted and instead an increase in the affinity of the high-affinity D2R agonist binding sites was obtained in the nucleus accumbens (Borroto-Escuela et al., 2018e).

Pathological changes in different types of heteroreceptor complexes may also develop in other types of brain diseases and work is ongoing. However, such vulnerable complexes still remain to be identified (Borroto-Escuela et al., 2017a).

Heterobivalent compounds with A2AR antagonists and D2R agonist pharmacophores are proposed to target A2AR-D2R heteromers and bring back D2R mediated transmission in part by the removal of the A2AR brake on D2R signaling. However, besides being large molecules with low brain penetration, they may not work in cocaine addiction. The reason is that the brake on D2R protomer recognition and signaling can no longer be significantly removed by actions at the orthosteric binding sites of the A2AR-D2R complex. Thus, only a minor component of the pathological A2AR-D2R heteromers in cocaine addiction may still respond to treatment with heterobivalent compounds due to marked and permanent deficits in their D2R protomer affinity and Gi/o mediated signaling.

## Current State of the Art. The Receptor Interface

In 2010, based on bioinformatic and mathematical approaches, it was proposed that a set of amino acid homologies present in both receptor interface surfaces within the heteroreceptors may have assisted in the formation of the complex (Tarakanov and Fuxe, 2010, 2013; Tarakanov et al., 2012). The theory was called the triplet puzzle. Three non-interacting sets of triplet homologies can be identified, namely protriplets, contratriplets and other triplets (Tarakanov and Fuxe, 2010). If a receptor pair has one or more protriplets but no contratriplets, a receptor heteromer is formed. In contrast, if a receptor pair has one or more contratriplets but no protriplets, the receptor pair becomes a nonheteromer. Interfaces are usually tight areas with complementary pockets distributed over the interface and characterized by the presence of conserved residues, for example, tryptophan and arginine. It was indicated (Bogan and Thorn, 1998) that hot spots of amino acids that bind each other and participate in the formation of the receptor interface. They are often surrounded by amino acids that isolate the hot spot from the solvent in this region. It should be considered that this may be the case in the amino acid protriplets in which one homology of amino acids form a hot spot by binding to each other, while the other two amino acid homologies are involved in isolating the hot spot from the surrounding solvent (Borroto-Escuela et al., 2018c). The protriplet amino acid homologies were originally proposed to be adhesive guides (Tarakanov and Fuxe, 2010) since they were also found to be the same like those in cell- adhesion receptors of marine sponges known to be highly conserved. However, in view of the above, it seems likely that protriplet amino acid homologies have additional functions such as forming hot spots that are isolated from the solvent.

### Mapping of the Receptor Interface

Of high significance for the field of oligomeric GPCR complexes is the mapping of the interface of the GPCR dimer studied since it will allow the development of interface-interfering peptides that specifically disrupt this GPCR homo or heterodimer. By microinjecting this interface interacting peptide into a discrete brain region, the function of this dimer can then be determined in this area (Borroto-Escuela et al., 2012, 2018e). A structural model of the A2AR-D2R heteromer was built in view of its clinical relevance for cocaine use disorder, Parkinson’s disease and schizophrenia (Borroto-Escuela et al., 2010a,b, 2018b).

The field was advanced by moving into GPCR crystal structures. It allowed the generation of atomic resolution models for receptor homodimers and heterodimers. In fact, crystal structures of A2AR (Sun et al., 2017), D2R and D3R (Chien et al., 2010; Wang et al., 2018) were recently determined. It was also found that several class A GPCRs can crystallize in the form of homodimers (Murakami and Kouyama, 2008; Wu et al., 2010; Granier et al., 2012; Manglik et al., 2012; Huang et al., 2013). It helped to understand how two GPCR monomers can orient towards each other. Atomic resolution models of A2AR-D2R heterodimers were obtained through experimental and computational work. Interface regions were identified using synthetic peptides which correspond to the seven transmembrane helices of the A2AR and D2R and their effects on the interface studied in BRET and PLA assays. Molecular dynamics (MD) simulations were used to refine the predicted heterodimer structure. Mutations were also predicted and were made to evaluate the model (Borroto-Escuela et al., 2018b).

Through this approach, it became possible to generate a structural model of the A2AR–D2R heterodimer. The primary interface appeared to involve TM-IV/V for both the A2AR and D2R protomers (Borroto-Escuela et al., 2018b). However, interactions with TM-VI may also participate in a dynamic way. These results are in agreement with the findings of the Franco group (Canals et al., 2003). It is important to underline that the C-terminal of the A2AR and IL3 of the D2R were not included in our heterodimer model in view of the lack of templates that can predict these regions. The strong electrostatic interactions between these two intracellular regions play a significant role in the A2AR-D2R interface (Ciruela et al., 2004; Woods et al., 2005; Borroto-Escuela et al., 2010a, 2011a).

The question is if we can improve our molecular understanding of the antagonistic allosteric A2AR-D2R interactions through the A2AR-D2R interface obtained in our model in the TM regions (Borroto-Escuela et al., 2010b, 2018e). This seems possible since the activated A2AR may reduce D2R agonist binding in part *via* an inward shift of TM-V of the D2R. However, this was so far only demonstrated with monoamine receptors homologous to the D2R but not with the D2R itself. Another important result is that the A2AR-D2R heteromers have a different interface from the A2AR homomers which makes differential targeting of either the A2AR homomers or the A2AR-D2R heteromers by means of interface-interfering peptides possible. Thus, the A2AR TM-IV and A2AR TM-V selectively blocked A2AR-D2R heteromerization vs. A2AR-A2AR homomerization (Borroto-Escuela et al., 2018e).

## Homo- and Hetero-Receptor Complexes and Their Allosteric Receptor Interactions in the Plasma Membrane Provide a Molecular Basis of Learning and Memory

When we introduced the concept of allosteric intramembrane receptor-receptor interactions in heteroreceptor complexes in the 1980s, there was little interest in this concept (Fuxe et al., 1983). The reason was mainly that there was no need for integration of signals in the plasma membrane since the intracellular integration of signals in the cells was sufficient. This did not make any sense for us for several reasons. In our minds, something additional should be going on to make use of the tremendous surface created by these networks.

With the findings of intramembrane receptor-receptor interactions in receptor complexes, it became clear to us that this huge plasma membrane area was used for molecular integration of receptor signaling by formation of receptor complexes from dimers to higher-order oligomers. The receptor protomers, as well as other proteins, could talk with each other *via* direct receptor-receptor and receptor-protein interactions through allosteric mechanisms. Then the signal integration continued in the multiple intracellular pathways.

The importance of this first step of information handling in the plasma membrane became clear to us early on. We introduced the allosteric theory of learning and memory based on the existence of many oligomeric receptor complexes in the postsynaptic and extra-synaptic membranes and the allosteric receptor-receptor interactions within them (Fuxe et al., 2014b,c; Borroto-Escuela et al., 2015a,b, 2016a).

Upon a change in the release pattern of transmitters in the synapse, learning of this new pattern will take place in the postsynaptic membrane through a reorganization of the homo and heteroreceptor complexes (Fuxe et al., 2014c; Borroto-Escuela et al., 2015a). Changes will also take place in the presynaptic receptor complexes to facilitate the maintenance of the pattern of multiple release of transmitters to be learned. There exists a kind of “basal barcode” of homo and heteroreceptor complexes. Through a transient reorganization of the receptor complexes a new barcode is obtained representing a short-term memory. A long-term memory is formed through the transformation of intracellular parts of the heteroreceptor complexes into soluble molecules that can bind to transcription factors and modulate their transcriptional actions at the DNA level. In this way, novel and specific adaptor proteins can be formed that bind to the receptor complexes short-term memory. Through the adaptor proteins the short-term memory becomes consolidated into a long-term memory (molecular engram) with conserved receptor-receptor and receptor-adaptor protein interactions. The adaptor proteins may act by increasing the links between receptor protomers themselves, receptor protomers and cytoskeletal proteins, and receptor protomers and scaffolding proteins (Fuxe et al., 1983, 2014a; Borroto-Escuela et al., 2015a, 2017a).

Such adaptor proteins have not yet been identified. However, it was recently possible to demonstrate that an activity-regulated cytoskeleton associated protein (Arc), which is a memory-related protein, can be formed (Okuno et al., 2012). In contrast to our postulated adaptor proteins, it was shown to weaken inactive synapses. The reason was that it had a high affinity for binding to the inactive form of calcium/calmodulin protein kinase II beta in inactive synapses, leading to the removal of the AMPA receptors. As a consequence, the contrast between active and inactive synapses is increased which can favor learning and memory.

Of high significance is the work of Everitt and colleagues (Milton and Everitt, 2012; Everitt, 2014) who hypothesized that drug addiction is caused by a pathological memory, called drug memory. They also proposed that understanding its molecular basis could lead to the introduction of novel anti relapse therapies. Our hypothesis on the molecular basis of learning and memory is in line with their view. We suggest that drug memories can be produced through a reorganization of the homo and heteroreceptor complexes in synapses and their extra-synaptic regions inter alia in glutamate synapses on the striatopallidal GABA anti-reward neurons (Borroto-Escuela et al., 2018c,d). Specifically, support has been obtained for the view that cocaine can produce pathological A2AR-D2R-Sigma1R complexes in such synapses. It appears to represent a long-term memory with a permanent and strong inhibition of D2R affinity and signaling which may lead to cocaine addiction. Therefore, A2AR-D2R-Sigma1R complexes can become a new target for the treatment of cocaine addiction (Borroto-Escuela et al., 2018d).

## Highlight of Future Directions

The current model of the A2AR-D2R heterodimer with a TMV-TMIV interface illustrates the importance of receptor-receptor docking and molecular dynamic simulations combined with biophysical techniques to map the receptor interface. By determining the interface of any receptor heteromer with these approaches it becomes possible to develop interface-interfering peptides that selectively disrupt the receptor heteromer studied. With interface-interfering peptides, it will also be possible to determine which effects on receptor recognition, signaling and trafficking are produced by allosteric receptor-receptor interactions and which are related to integration in the intracellular signaling pathways.

Super-resolution imaging is a method that will give new insights into GPCR oligomerization at the single molecule level (Jonas et al., 2015, 2016). It involves the use of photoactivated localization microscopy in combination with photoactivatable dyes. The GPCR monomers, dimers and oligomers can be visualized to a resolution of less than 10 nm and quantitation can be obtained through the analysis of data sets from these types of complexes. This method is just at its beginning and will be useful in the field of GPCR homo- and heteromerization.

Spatial intensity distribution analysis is another novel promising method of significance to determine if GPCRs exist as monomers, dimers and/or higher-order oligomers (Ward et al., 2015). Through this method, it is possible to obtain densities of molecules fluorescence including their quantal brightness (Barbeau et al., 2013). With this analysis, it became possible e.g., to indicate that 5-HT2C receptors exist as a mixture of monomers, homodimers and higher-order homomers which becomes dominated by monomers after treatment with 5-HT2C antagonists (Ward et al., 2015).

This field of molecular integration illustrates the high impact of the heteroreceptor complexes and their allosteric receptor-receptor interactions on the regulation of brain networks (Borroto-Escuela et al., 2017a).

## Author Contributions

We confirm and declare that all authors meet the criteria for authorship according to the ICMJE, including approval of the final manuscript, and they take public responsibility for the work and have full confidence in the accuracy and integrity of the work of other group authors. They have substantially contributed to the conception or design of the current review. They have also participated in the acquisition, analysis and interpretation of data for the current review version. Furthermore, they have helped in revising it critically for important intellectual content and provided final approval of the version to be published. In addition, they have contributed in this last version of the manuscript to writing, technical editing and language editing.

## Conflict of Interest

The authors declare that the research was conducted in the absence of any commercial or financial relationships that could be construed as a potential conflict of interest.

## Footnotes

^1^www.gpcr-hetnet.com
